# Enhancing research culture through PhD training: a systems approach to identifying leverage points for policy formation

**DOI:** 10.12688/wellcomeopenres.19567.1

**Published:** 2023-09-25

**Authors:** Rebecca Martin, Nazia Nasir, Annamaria Carusi

**Affiliations:** 1 Centre for History in Public Health, London School of Hygiene and Tropical Medicine, London, UK; 2 UK Research Development, Research and Innovation Services, Univresity of Leeds, Leeds, UK; 3 Interchange Research, London, UK

**Keywords:** Research culture, PhD supervision, research funding, graduate training policy, systems approach

## Abstract

This article examines the role of PhD training programmes in identifying and implementing positive interventions in research culture in the biosciences. Using a data set consisting of transcripts from interviews and group discussions with 179 participants from 18 of the current 23 (78%) UK-based Wellcome-funded PhD programmes, we apply a systems theory methodology to the system of higher education and PhD training. Using system mapping as an investigative tool, this approach identifies points of leverage within the system where policy interventions might be best targeted to affect changes to research culture in the global higher education sector. The results of this investigation highlight the student-supervisor relationship as a nexus for these interventions and recommends the programme structure as a global policy for PhD training.

## Introduction

Research culture in academic institutions is today widely recognised as being ‘toxic’ ( Kent, 2019); many issues have been identified, especially over the last decade, including problems with personnel management, work-life imbalance, bullying and harassment, ‘stifling’ publication culture, a prevalence of stress and poor mental health, and a lack of diversity ( Arnette, 2003; Christian *et al.*, 2021; Dhirasasna *et al.*, 2021; Mudrak *et al.*, 2018; Van Noorden, 2018; Wellcome and Shift Learning, 2020; Woolston, 2019b). *Nature*’s 2019 PhD Study found that 36% of PhD students and 33% of the academic staff had sought help for anxiety or depression for research work associated stress ( Woolston, 2019b). Disadvantaged groups (female, non-binary, LGBTQ+, and disabled researchers) were significantly more like to have sought professional help for anxiety or depression. 76% of students reported working for more than 41 hours per week. Meanwhile, 61% of academic staff have witnessed bullying or harassment, with 43% experiencing it personally ( Wellcome and Shift Learning, 2020).

However, it is not clear how best to improve research culture through interventions on a policy level. That is not to say that there is nothing being done in this area; on the contrary, there are various initiatives aiming to tackle research culture issues ( Raymer, 2019). For instance, there are initiatives aimed at reducing over-reliance on metrics and research quantity in staff evaluation, for both promotions and grant applications, including the introduction of narrative CVs and a concordat to support the career development of researchers ( Catlow, 2019; Lacchia, 2021; Woolston, 2019a; Redden, 2019; Saenen *et al.*, 2021; Schmidt *et al.*, 2021). There are also a number of recent Equality, Diversity, and Inclusion initiatives which aim to increase the diversity of the workforce to the benefit of science, for example the introduction of the Race Equality Charter in 2016 in British higher education (HE) ( AdvanceHE, 2019; Engineering Council, 2022; Wade *et al.*, 2019). As these are recent initiatives, it is premature to expect clear evidence of success or otherwise. However, many of these initiatives tackle isolated research culture issues, whereas our work looks to identify points of leverage which have the power to change the system as a whole.

In 2018, one of the UK’s largest funders of biomedical research, Wellcome, reviewed their PhD training programmes ( Wellcome, 2018). A key finding of the review was: ‘PhD training could be improved to support a more positive research culture. We want to redesign our PhD programmes so they combine scientific excellence with a commitment to improving the working environment for students.’ Wellcome thus decided to intervene at the level of PhD training, for the first time in the history of its funding at this level, giving positive research culture the same priority as scientific excellence in the evaluation of applications ( Coriat, 2019). Positive research culture was not defined conceptually in advance; rather the approach of definition by examples was used. The main examples of the aspects of PhD training that fall under research culture were given as: recruiting for diversity and promoting diversity across all levels of the PhD; support for mental health and well-being; a commitment to end bullying and harassment; a promotion of good research practice and integrity; student-centred training; and support for career progressions including outside of academia. Proposals could also include other themes that they saw as relevant to research culture. This paper is a reflection on the early outcomes of this programme, working with a community of practice consisting of the members of all 23 PhD programmes funded under this new iteration of the scheme.

The research for this paper focuses on three areas of PhD training: supervisory practices, mental health and wellbeing, and working hours and locations. Using a systems theory approach, we show how intervening in research culture *via* PhD training affects institutions and processes through which research culture is enacted more broadly. Taking this systems approach to understanding research culture, rather than tackling isolated issues, we aim to join up thinking across multiple research culture issues, and provide suggested areas for policy action where further positive changes can be made that will reverberate throughout the system.

This paper first explores the utility of systems theory for approaching the improvement of research culture in academia. We then go on to discuss our methodology: a participant action research approach within a community of practice. The main section of the paper considers the way agency and power interact within our three key areas of the academic system – working hours and locations, mental health, and supervisory practices – mapping these networks of agency visually. We then discuss how, through this mapping, we have been able to identify the student-supervisory relationship as an optimal leverage point for policy intervention, demonstrating that this relationship forms the basis of a strong feedback loop with potential to affect many different levels of the system.

## Literature review

### Research culture

Research culture is a concept still in flux. It has been variously defined, and different institutions and individuals take the term to refer to a wide variety, and indeed different combinations, of parts of academic life. The Royal Society defines research culture as: ‘... encompass[ing] the behaviours, values, expectations, attitudes and norms of our research communities. It influences researchers’ career paths and determines the way that research is conducted and communicated’ ( Royal Society, 2018). While many organizations, like Science Europe, have adapted this definition ( Science Europe, nd.), others like the Wellcome have focused on understanding what the current research culture looks like to the researchers ( Wellcome and Shift Learning, 2020). Others describe research culture as a ‘hazy concept, which includes the way we evaluate, support and reward quality in research, how we recognise varied contributions to a research activity, and the way we support different career paths.’ ( Casci & Adams, 2020). Meanwhile, Evans (2007) presents a working definition of research culture as ‘shared values, assumptions, beliefs, rituals and other forms of behaviour whose central focus is the acceptance and recognition of research practice and output as valued, worthwhile and preeminent activity’ (p. 2). Similarly connecting research culture with research output, Akerlind (2008) defines research culture as ‘enhancing one’s capacity to contribute to a larger field or community’ (p. 253). Through our research we have examined what members of our community of practice take research culture to mean, with participants variously describing research culture as either the context in which research currently takes place, or the ideal conditions under which research could take place ( Carusi *et al.,* 2022b). This lack of consensus on the basic terminology of the topic of study necessarily creates divisions within the literature.

The main characteristics of research culture are often linked with productivity or capacity, conceived as part of the marketisation and neo liberalization of HE, including the pressure to publish and to demonstrate the public utility of academic research, as well as the pressure to perform additional public service (US) or impact (UK) work ( *e.g.* Ball & Crawford, 2020; Ion & Castro Ceacero, 2017; Khoo, 2021; Roberts, 2007; Thornton & Jaeger, 2008). ^1^ Some scholars have investigated the impact of new research assessment frameworks on research culture ( Billot & Codling, 2013; Middleton, 2005). Meanwhile, others have begun to outline the understanding and perception of this new research culture from the perspective of academics within the field ( Holligan *et al.*, 2011). 

In a survey by Wellcome and Shift Learning (2020) more than half the 4,200 responders used negative words to describe research culture, with experiences of exploitation, discrimination, harassment and bullying. Researchers felt poor research culture leads to stress, anxiety, mental health issues as well as strained personal relationships, and noted feelings of isolation and loneliness at work. Only a third of the 3,200 active researchers felt secure pursuing a career in research. About half of the respondents who had left the research community cited difficulty in finding a job and an insecure career path as one of the reasons for their career switch. The survey concluded that a cultural change would require research culture to be creative, supportive and collaborative. Publications focusing on research culture in established research settings also argue that it is now necessary to prioritise quality over quantity in academic settings, which includes taking care of the physical and mental wellbeing of researchers ( Fischer *et al.*, 2012; Harvey, 2020). Our research contributes to these understandings of research culture, focusing on how it is perceived and experienced by students and staff.

### Systems theory

**Figure 1.**
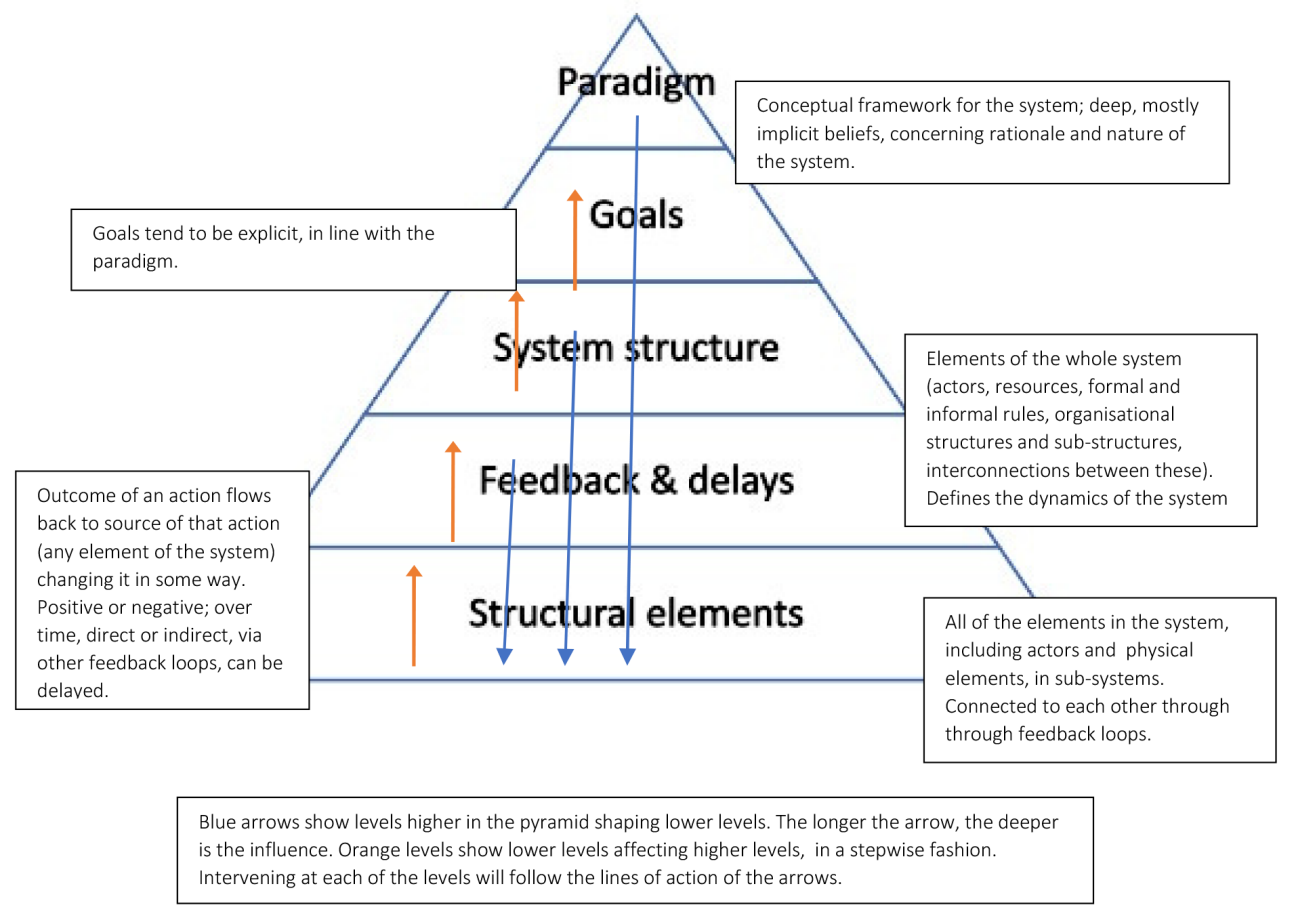
Five Intervention Levels. Adapted from Malhi *et al.,* 2009, and Meadows, 2008.

Systems theory is an approach that is increasingly used to analyze and inform policy and other interventions in many different areas, such as health, nutrition, and HE ( Bawden, 1991; Ison, 1999; Peters, 2014). Donella Meadows (2008) describes a system as an interconnected set of elements that is coherently organised in a way that achieves something. Understanding the elements, boundaries, and relationships within a given system is vital for identifying the points at which policy change might be most effective and impactful ( Dattée & Barlow, 2010). Holmes *et al.* (2012) discuss the factors that make a system complex rather than complicated, including the heterogeneity and interdependence of structural elements, the non-linearity of causation, the existence of feedback loops, and the dynamic nature of processes, and emergent nature of behaviours. They use the systems approach to show how the system can be changed through intervening on specific leverage points or levels. Following Meadows’ initial 12 levels at which interventions can be made to change the system, they distinguish five levels (see Figure 1). 

In the context of research culture and HE, Dhirasasna *et al.* (2021) found a systems approach appropriate for analyzing students’ wellbeing as it ‘comprises several interactive components whose relationships are inherently non-linear’. They also noted systems theory’s inclusion of feedback mechanisms in complex systems that shape wellbeing. Gibbs and Griffin (2013) concluded that social identity, and structural dynamics of the biomedical workforce played an important role in career aspirations of PhD students, and retaining a diverse talent required institutional and systemic reforms. Another area of research culture scholarship that has been analyzed using systems theory is research assessment. Lee *et al.* (2021) have used a systems approach to analyze and design interventions, noting that ‘Systems thinking can provide insight into areas where institutions have the highest chances of making strategic interventions, with the goal of advancing practical and robust approaches to research assessment globally and across scholarly disciplines.’ This has contributed to the Declaration of Research Assessment (DORA’s) understanding of how to change practices of research assessment, which deeply affect research culture. DORA and Howard Hughes Medical Institute (HHMI) also convened a diverse group of stakeholders to explore different approaches to cultural and systems change to improve research assessment policies and practices ( DORA Meeting, 2019). Similarly, we maintain that understanding the way that PhD training sits within research culture from a systems perspective enables us to get a clearer view of the interacting components, the feedback loops in which they are embedded, and how strategies for intervening on research culture – starting from training but potentially more broadly in the research system – could be designed. 

Our research has been conducted with a community of students and staff of PhD training programmes funded by Wellcome in an intervention that aimed to give equal priority to research culture as to scientific excellence. Wellcome did this by asking for proposals that would show how PhD training would be offered in a student-centred way. This was an intervention in the delivery of PhD training, aiming to bring about a change in the culture of training, as well as in research culture more broadly. This paper reflects on how that intervention has played out in the system of PhD training as it is situated within a broader context of research culture. We use the systems approach in order to identify the elements of the system – in our case, focusing on agents – and the interconnections between them. We aim to show that the intervention by the funders has provided a platform for adding a feedback loop from students to supervisors in a way that potentially changes the system structure, which in turn could feed into changing the understanding of the goal of the system (see also Kim, 1999), and the paradigm of beliefs and expectations about PhD training, and related aspects of research culture. However, in line with all of these systems thinkers, multiple interventions at multiple levels are needed in order to sustain this feedback loop and, in view of the expected delays within feedback loops, time plays a key role in establishing feedback loops and understanding their effects.

## Methods

### Ethics

The research for this paper obtained ethical approval, on 04/12/2020 from an ad hoc panel comprised of three ethicists, *via* the Social Research Association, in view of Interchange Research Ltd. not having its own review board. Participants gave written, informed consent prior to commencement of the study.

### Study participants

The research for this paper was conducted within a community of practice consisting of students, academic staff, and professional services staff who are part of Wellcome-funded PhD training programmes. ^2^ The community of practice focuses on the research culture component of these training programmes, and was established to provide a platform that would enable those involved in the programmes to gain a better understanding of research culture, and to reflect on and share their practices and experience relating to research culture. The role of the authors of this paper in the community of practice was to undertake a form of participant research.

### Choice of topics

The most recent round of Wellcome funded PhD training programmes were running for only a few months before the COVID-19 pandemic hit. As the community of practice was only just established at this time, our initial focus shifted towards considering the impact of the pandemic on research culture and what could be put in place to maintain the improvements prompted by, and mitigate the negative effects of, the pandemic. We hosted two virtual discussion sessions with both staff and students to investigate their experiences of the pandemic. Apart from revealing the understanding shown by students of the struggles faced by staff, and vice versa, what emerged from these discussions was that the pandemic had highlighted and exacerbated a lot of pre-existing issues within the academic system. These discussions are summarised in a report titled *Talking About Research Culture: A report on discussions about research culture in Wellcome Trust PhD programmemes* ( Carusi *et al.,* 2022a). These two sessions revealed four areas in which participants felt more action was needed to help improve research culture.

These four action areas were:

1. Addressing the ongoing negative impacts of the pandemic on researchers’ funding and career prospects
2. Identifying and supporting good supervisory practice
3. Negotiating flexible working hours/locations
4. Emphasizing mental health as an aspect of research culture

We used these four action areas as a springboard for our next round of community of practice discussions ( Carusi *et al.,* 2022b). These were organised at each individual programme, leaving it up to them to decide who would be included within their own institutions. Group discussions were held at 18 out of the total 23 programmes. These were mostly held online, using Zoom, with only two hosted in-person (due to COVID-19 related restrictions). The focus of our research in this instance was determined by what the community wanted to discuss. ^3^ The topics in order of preference for discussion were two, four, three, and one. There is not a separate section for topic one, but the observations relating to it are interspersed in the other sections (see Carusi *et al.,* 2022b) for a detailed treatment of the topic). 

### Facilitation of group discussions

We approached each discussion as an opportunity for participants to articulate their experience of a topic and also what actions or interventions they believe are necessary to bring about a positive change. Our action research approach is informed by the view of systems thinking as ‘an iterative learning process’ ( Sterman, 2006), in that we adjusted the facilitation of each group discussion as we learned about the system, asking later groups open questions about topics raised in earlier groups to either corroborate or raise further questions about their understandings of the system. We found a huge amount of consensus across the groups. In this way our process was both iterative and corroborative, allowing us to build a much firmer sense of the system in which the action areas are embedded.

**Figure 2.**
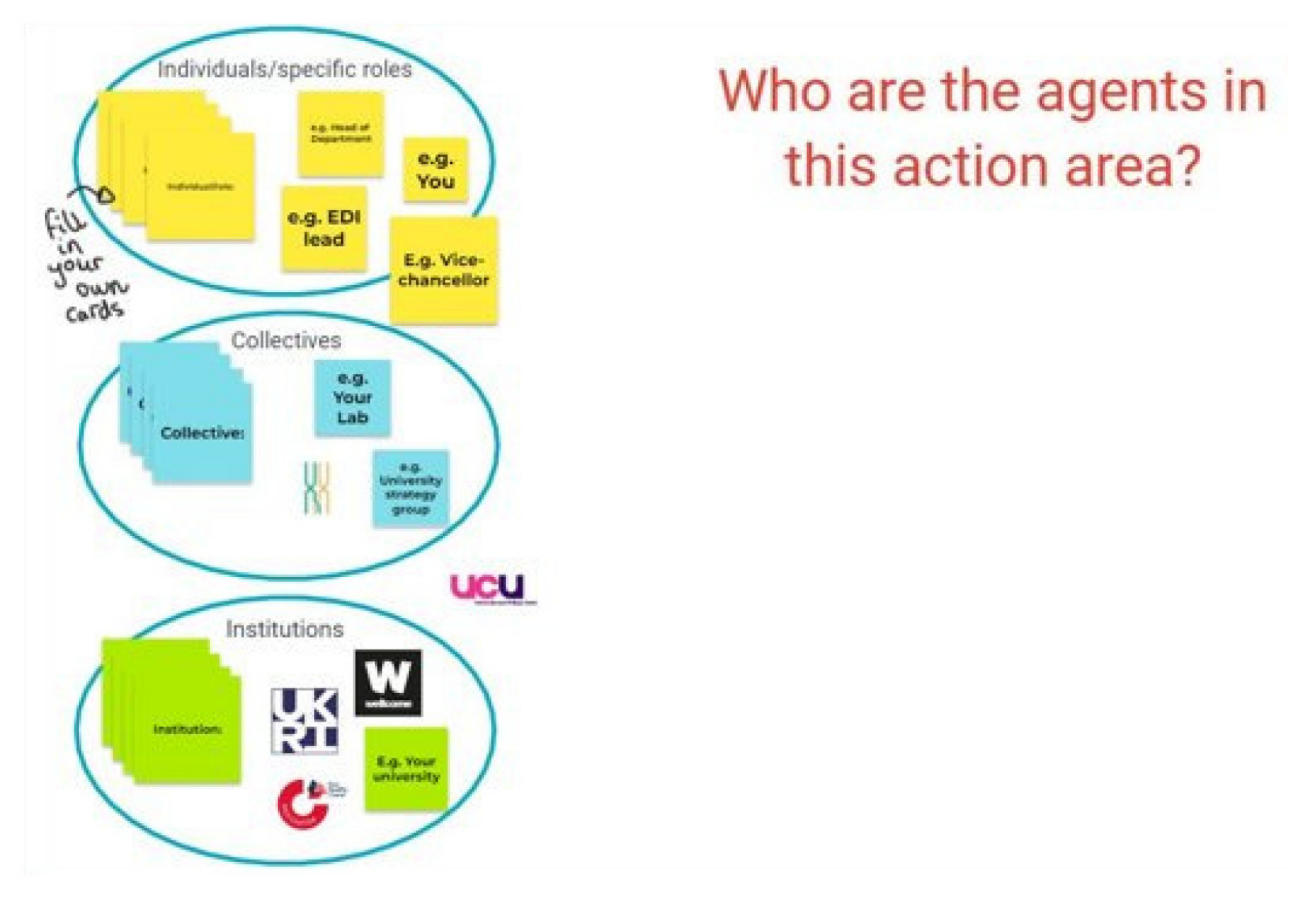
Sample pre-populated map of agents provided to the participants using the Google Jamboard. The agents were sorted under different groups of individuals, collectives and institutions.

**Figure 3.**
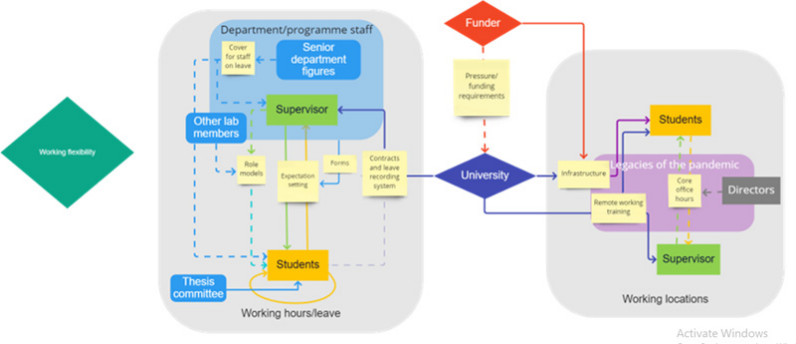
The map of agency generated for the topic ‘Working hours and location’. The narrative key has been explained in the text.

Bringing ‘engagement between people’ ( Willis *et al.*, 2014) to the fore of our own methodology, we began our group sessions by asking our participants to map networks of agency with respect to a particular action area to aid discussion. For this task we asked each group to choose the action area(s) that they felt most important to discuss, using a poll to gauge the interest in each area. The maps were pre-populated with some suggestions from the research team, which were divided into groups of agents in line with the common tripartite institutional studies framework: individuals, collectives, and institutions ( Giorgi *et al.*, 2015) (see Figure 2). The participants were actively encouraged to come up with their own agents and to question the groupings provided, which some did. Participants added to the maps using either virtual or physical post-it notes to identify the agents with the power to make change in their chosen action area(s), whilst discussing in further depth their potential roles and the relationship between these agents. However, groups engaged with the physical mapping element of this task to varying degrees, with some discussing the topic of agents and agency rather than adding to the map directly. As such, we have collated data from both the maps and discussions across the 18 sessions for our analysis (see Figure 3– Figure 5).

**Figure 4.**
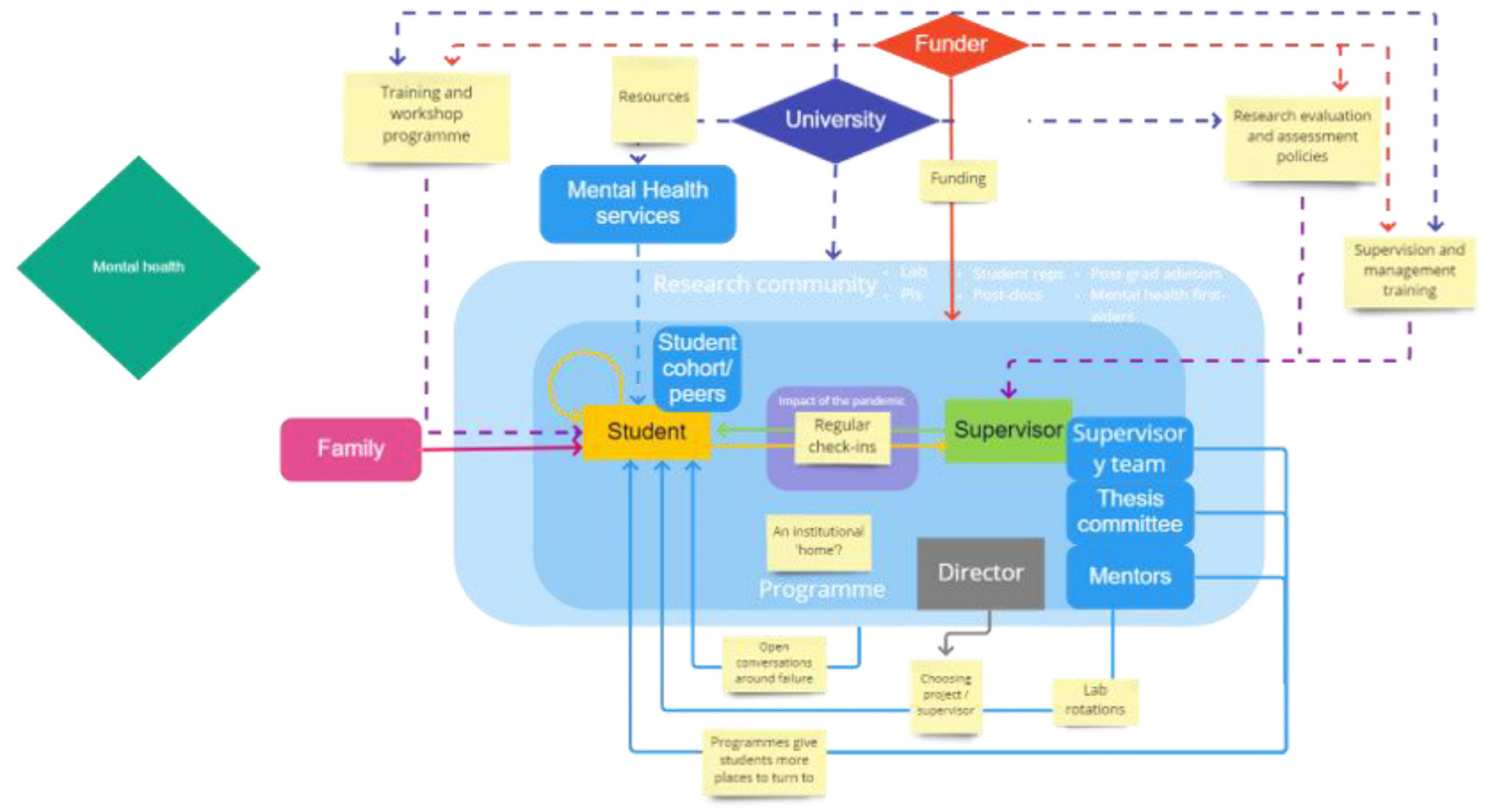
The map of agency generated for the topic ‘Mental health’. The narrative key has been explained in the text.

**Figure 5.**
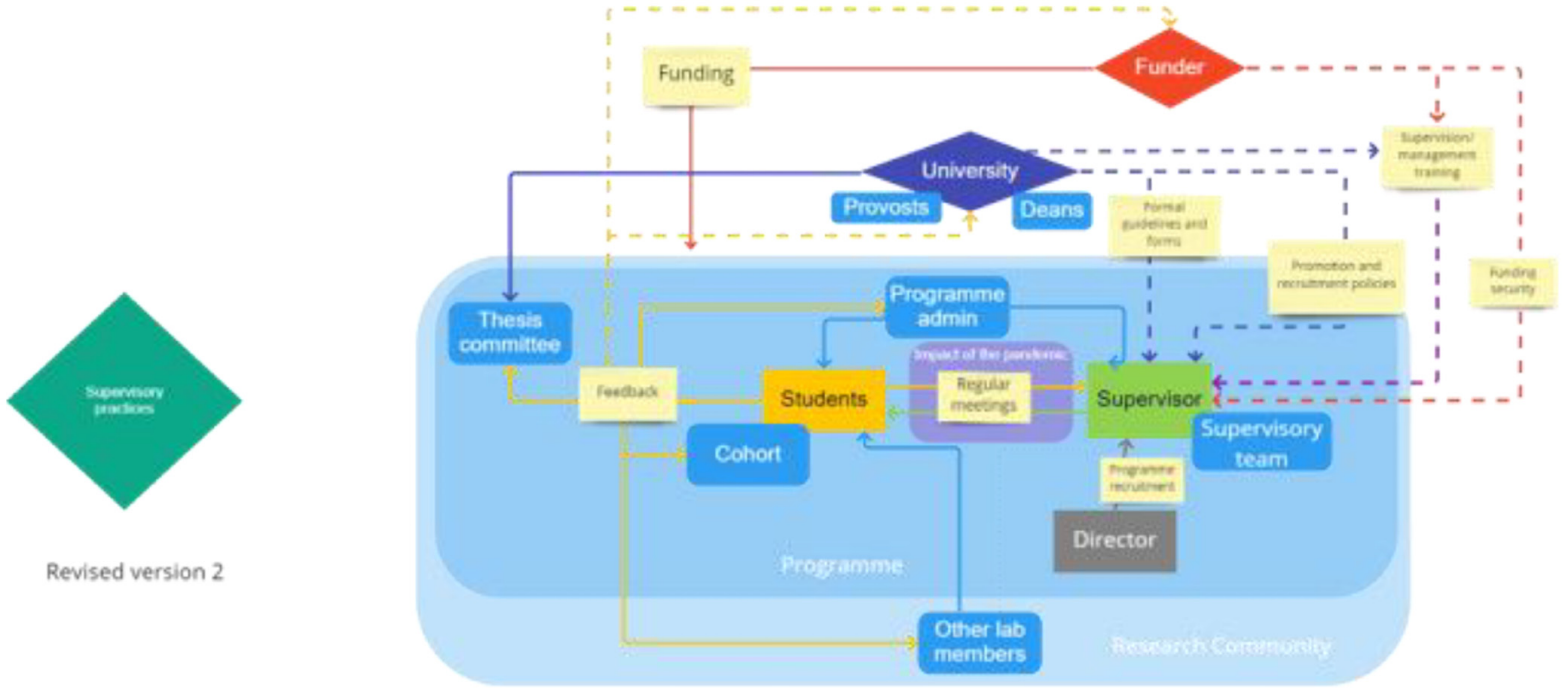
The map of agency generated for the topic ‘Supervisory practices’. The narrative key has been explained in the text.

### Interviews

Alongside these discussions, we also held interviews with the programme directors, mostly one-on-one with one of the main directors, but sometimes with more than one director or with teams including programme managers or coordinators. Each programme decided who to put forward for these interviews, including in each case at least one director or co-director. Again, it was possible to arrange directors’ interviews with 18 of the 23 Wellcome Programmes, with the same programmes that participated in the group discussions. These were semi-structured interviews, exploring the agency Directors, and other staff who are key to delivering the programmes, perceived themselves to have in the context of their institutions (see *Extended data*, ( Carusi *et al*., 2023)).

The directors’ interviews focused largely on three topics. The first was on the topic of diversity in student recruitment, an important topic which will be discussed in the context of a broader study of recruitment across the Wellcome programmes. The second and third topics, which informed the work of this paper, were supervisory practices and the role of the programmes within institutions. The former helped us to elucidate supervisory practices from the perspective of staff, whilst the latter helped outline the influence of programmes within their universities, revealing the changes that they are able to instigate outside of their own programmes. The contents of these interviews have been summarised in an independent report ( Carusi *et al.*, 2022c).

Both the group discussions and directors’ interviews were opportunities for participants to express their experiences of the research system and, through dialogue with each other, explore the topic from different perspectives. The maps reflect the views of the action areas that emerged from these dialogic conversations.

### Data recording

We recorded all the group discussions and interviews using Zoom (when online) and using mobile devices (when in-person). All recordings were converted to text using an automated transcription tool, Otter. Once completed, we manually went through the transcribed files and the audio recordings, made corrections and anonymised the data.

### Data analysis

We conducted a qualitative thematic analysis of the transcriptions. In line with the agency-based facilitation structure used in the group discussions, and the agency-based line of questioning in the structured interviews, our analysis focused on agency. Specifically, we explored who has agency, which actions these agents were able to undertake, and who is affected by these actions. In addition, we queried how agency could be further developed, or which further actions could be undertaken, and which goals this would achieve. With respect to the focus on agency which framed the facilitation of discussions, our analysis was deductive, or top-down as we used the categories of agency for analysis ( Braun & Clarke, 2012); in all other respects, our analysis was inductive or grounded, looking for associated themes as they emerged ( Braun & Clarke, 2006). The themes we have picked out are not based on quantitative analysis, but on the significance of the theme in a pattern or set of relationships, based on patterns of meaning, especially relationships among agents (see *Extended data,* ( Carusi *et al*., 2023)). 

### Constructing maps of agency- identifying agents and their relationships

The main focus of this paper is to understand agency in PhD training, in order to shed light on the extent to which those delivering or receiving training felt themselves acting or able to act. This could be explored from multiple perspectives, and generally the questions we asked were: who influences or shapes the area (that had been chosen), and who is able to change it? Both the group discussions and directors’ interviews were opportunities for participants to express their experiences of the research system and, through dialogue with each other, explore the topic from different perspectives. The maps reflect the views of the action areas that emerged from these conversations.

The predominant view that emerged from these conversations with participants is that each action area is characterised by a network of highly interconnected agents, with actions that ripple out beyond their immediate targets, in complex feedback loops. To gain a more detailed understanding of the systems that influence research culture and to elucidate the complexities of the system, we have mapped out the agents and relationships highlighted during our discussions for three of the four action areas- working hours and location, mental health, and supervisory practices. The ongoing impact of the pandemic received much less attention in these later discussions and has been integrated into the other three maps, illustrated by a purple box.

To develop the maps of agency ( Figure 3– Figure 5), we first noted (non-quantitatively) all the agents against the action points that came up during group discussions from each group of individuals, collectives, and institutions. We then arranged each agent on the map to allow plotting of each mentioned connection and relationship between agents in a clear manner, presenting the individual agents, such as students and supervisors, or collectives, such as supervisory teams, as a part of the larger system of PhD training. In our interpretation of the data, we also noted which actions affect a relationship.

These maps reveal that agents are typically acted upon themselves as much as they act upon others and constrain as much as they are constrained by others, giving rise to feedback loops. In addition, we found that all three topics were highly interconnected. This was clear from the discussions as, no matter which topic our groups started with, they often went onto include elements of or fully embrace at least one other topic by the end of the short sessions.

## Results

### Reading the maps: a narrative key

It is necessary to provide a brief explanation of how to read the maps which now follow in this section. Firstly, we have used different shapes to represent different kinds of agents: rectangles with sharp corners show individual actors, whilst those with rounded corners are collectives or undefined individuals; institutions are represented by a diamond shape. Each of the five main agents identified has their own color, with correspondingly colored arrows: universities are blue, funders are red, supervisors are green, programme directors are grey, and students are yellow. Meanwhile, light yellow boxes are used to show a key action that occurs, or could occur, along a line of connection between two actors. Where two agents feed into one action, the two colors are blended to create the line from the action to the receiving agent. For example, where both universities (blue) and funders (red) have influence over influence funding and promotion metrics, the resultant line from the action to the agent is purple.

These maps also reflect the areas of the system that members of the community of practice participating in these discussions perceived as requiring change. Solid lines or arrows signify that a process that is currently in place and perceived to be working effectively. In contrast, dashed lines or arrows illustrate either that participants felt a new connection should be established or that an existing one needs to be improved. A dotted line or arrow is used only once in these maps to indicate the temporary shift of a role from one agent to another, specifically during the COVID-19 pandemic. These lines were constructed solely on the observations on our research participants. As such, a solid line does not show that there is no possible work to be done in a particular area or interaction, but only that our participants were satisfied with the current condition of this relationship.

Each action point is complex and that change at these points can have both positive and negative effects on the actors within these maps. For example, contracts can provide clarity but also be perceived as restrictive. As such, there should be careful discussion around changes to be made on any of these action points, where change is perceived necessary, to ensure a balance between these kinds of considerations.

### Map 1: working hours and locations

Working hours and locations came to the fore as an issue in our initial discussions on the impact of the pandemic particularly because, during the time of the strictest Covid-related restrictions, working from home meant that there was a greater blurring of lines between work and home than is normally characteristic of academic work ( Carusi *et al*., 2022a). This gave rise to varied and sometimes conflicting reactions. Many staff and students reported high levels of stress and anxiety resulting from the absence of work/life balance or division between work and domestic tasks. However, at the same time, many (sometimes the same individuals) also expressed a desire to continue hybrid working. The management (either explicit or implicit) of where and when academic work should be done is clearly an important channel through which hierarchies of power and agency are exercised. 

From our discussions, it is clear that the expectation that academic scientists would work long hours and weekends, and sacrifice their personal life, for the sake of their research career is currently being re-evaluated. Voices that stressed the need for work/life balance and clearer boundaries between them were juxtaposed by voices that reiterated the need for long working hours for successful scientific careers. This debate was encapsulated in the metaphor of ‘Formula One science’ used by a member of academic staff at one of our group discussions:

‘ *So a pretty famous scientist, a while ago, said apparently to one of his postdocs that science is a little bit like these different tiers of racing. So there's Formula One, and there's also the local go-kart racing. They're both about racing, but they're not the same. And if you want to do Formula One, then the working conditions are very different.’*

Another member of staff pushed back on this concept, illustrating the tensions here:

‘ *And yet the ones who are in the go-karting league are the ones who are still going, right? And actually a few who were in the Formula One, absolutely, they're managing their own labs... they're on that high trajectory career path. They're happy, they're stressed, but they're happy. They're doing well in a competitive way. And the go-karting people are equally happy...the reality of it, though, they're both competitive leagues*.’

Still another member of staff linked this dilemma more explicitly to the mental health of researchers, noting particularly that ‘it takes strong mental health’ to be consciously in the ‘go-kart league’ when opportunities for comparison between peers arise so frequently on platforms like Twitter:

*‘If you're in the go-kart league, you have to be 100% happy about that. And you have to say, no, I am actively deciding to do it... to be in this place, because otherwise it's going to eat at you. It's going to eat at you and you're gonna say I should be doing Formula One. And that is the mental health thing that I see. And that is what kills people.’*

During the same discussion other participants questioned what was wrong with ‘go-kart science’ pointing out the advantages of taking this approach: *‘And I have seen people actually only do 40 hours a week in the go-karting league produce fantastic science. And, you know, basically do a great job.’* This was corroborated in other discussions, with a significant minority of the Directors interviewed questioning the expectation that one could not have a successful research career without sacrificing one’s life outside of work.

Most programmes reported that the University formulates the rules and guidelines surrounding working hours, annual leave, and working locations for staff. It was widely acknowledged that the enforcement of these rules and use of the leave recording system varies widely across universities and even within the same university or department. However, while staff therefore already have a system of leave recording in place, students are not considered employees and therefore do not have the same centrally instituted system in place. Many students felt that without such a system, it was not clear to them how much leave they are entitled to, or what their working hours actually are. They were also keen to exercise their own agency in setting the parameters of their working lives, and to have in place at least semi-formal systems for doing so, such as some type of recording system, which would allow them to take the leave they are entitled to. Some participants suggested a formalised contract or memorandum of understanding that could be negotiated at the start of the PhD. While in many cases, departments, or graduate schools, or similar university bodies for taking care of doctoral students, do have guidelines or procedures to ensure that students take leave, students noted that they either did not know about them or that they were often tick-box forms and were not properly followed up on.

With so much left to informal systems, the student-supervisor relationship has an important role in setting expected working hours and leave taking. As our map in Figure 3 illustrates, students pick up implicit practices from their supervisors, as well as other staff members, using these individuals as role models for how to structure their time. For example, if supervisors sent and responded to emails outside of normal nine-five working hours, then students reported feeling that they should do the same. A member of academic staff noted that: ‘Supervisor cognitive dissonance is a real issue in academia [where supervisors may be telling the students to take leave while] very clearly demonstrating to the student that actually they don't think they should be doing that’. As such, Figure 3 shows that this role modelling requires improvement through the use of dashed lines and arrows.

It was suggested that thesis committee members could play an important role in ensuring that students were working ‘normal’ working hours and taking adequate leave, through asking about this explicitly in review meetings. However, students still said they would hesitate before turning to their thesis committees for help on this as they felt committee members may share the same implicit expectations or be reluctant to appear critical of other supervisors. 

Despite the strong top-down influence of supervisors, students were conscious of their own agency over their time when making decisions to limit their working hours. For example, a student noted that ‘the more work you do, the more work you get to do […] but I think as a student, it’s also up to us to try to draw that line, where we say, okay, we’ll work within a certain limit […] and make it known to the supervisor, that this is, kind of, the line’. Students also felt responsible for self-regulating their working hours and leave to avoid burnout. This role of the student in their own self-regulation is illustrated by a closed yellow loop on Figure 3. Students did, however, feel that they needed to be given more agency to decide their working styles and more training or role modelling to help them build their own healthy work/life balance. 

Students felt that one particular situation which could have an immediate detrimental impact on work/life balance and working hours was when staff had to take unexpected leaves of absence. It was not the leave itself which concerned students, but rather the lack of cover for the missing staff member. This resulted in students having to take on more work, for example in supervising masters students, without any increase in the hours available to them or decrease in other work to be done. Student participants felt that cover for these kinds of staff absences should be taken care of by senior members of the department, suggesting that there needed to be better planning for these situations. This is illustrated by dotted lines in Figure 3, as both staff and students felt the need for improvement in this area.

Alongside working hours and leave, the location of work was also increasingly seen as flexible, with working practices during the pandemic having set a precedent for the amount of work that can be conducted away from the lab or the office. The University as an institutional agent was seen to influence working location flexibility by the infrastructure it puts in place for remote work. The role of a funder like Wellcome providing laptops to all students also marked out funders as an important enabling agent, allowing students to also work from home. As can be seen in Figure 3, staff did not discuss receiving resources from funders directly to facilitate remote working, although it is possible that there were resources available to them as a result of their funding, and thus resources are not seen to flow directly from funder to staff in our working hours and leave map. The major continued legacy of the pandemic was the adoption of more flexible working locations, although many wanted some overlap between the working hours and location of all lab members for the creation of community and knowledge sharing. One director pointed out that for the sake of team cohesion, people should be present at the same time at least sometimes. Directors of programmes or lab directors – who also have a team building role – have a strong role in defining what is acceptable in this area of working locations, often setting these times for in-person working.

The map of agency for this action area shows a top-down flow of agency in shaping the area and potentially changing it. Universities put in place the infrastructure for working hours, leave taking, and working locations. There is a perceived gap in this infrastructure when it comes to students’ hours and leave, leading to ambiguity and confusion, that allows informal and implicit expectation setting (for example through role modelling) to have a strong influence (dotted line on the map). Sometimes this gap was attributed to funders, with some participants saying that funders should set prerequisites for good working hours and location practices. Students’ requests for a more formal system of recording, or some means of making expectations on both sides explicit (such as a memorandum of understanding), seemed to spring from a desire to be able to exert agency in the relationship, and gain more control over work/life balance. However, it is reasonable to expect that improvements in this area would, in turn, lead to changes for supervisors too, at least with respect to expectations, and possibly in their own behaviour. In contrast, the working locations side of this map shows that resources from funders and universities, such as hardware and training, are both already in place. Here we can see there is much more room for negotiation between students and staff, including both directors and supervisors.

### Map 2: mental health

Mental health was the topic that received the most attention in our earlier Pandemic Experiences workshops ( Carusi *et al.,* 2022a), and was the second most frequent action area chosen in the subsequent group discussions. Mental health is a very broad area and can cover anything on a spectrum from equilibrium and contentment, to strains and stresses, to full-blown mental health crises. In our discussions we left it undefined, waiting to see how participants would pick up the term themselves. It was clear that when there was a need for Mental Health Services – such as counselling or therapy –participants perceived that most change and additional resources in this area needed to come from the University as an institutional agent (indicated by the dotted line in Figure 4). Mental Health Services currently provided by each university were discussed as helpful but frequently underfunded. Students noted that a lack of resources given to these services by the university usually meant that it was often very difficult to be seen and that sessions were limited, which could be actively detrimental in a crisis. Almost everyone – staff and students – said that they did not know what was available in their institutions in terms of mental health support, and that there was either lack of awareness, or lack of a centralised hub of information on what are often a range of dispersed services. 

However, it was also clear from the discussions that Mental Health Services play a peripheral role in the mental health action area, and that most of the concern about mental health is about the grey area before there is a need for Mental Health Services:

*‘In reality, it's just averting the crisis, getting it just a step away from the from the edge, and then calling it a problem solved. Rather there should be continued support by ensuring everyone's lives are enriched versus the symptom-treatment way of viewing mental health. If it's not at a breaking point, it's not a problem kind of attitude. The University mental health systems are designed to get you working again, rather than to get you better again’*

When asked to identify the agents who shape mental health as an action area, students often mentioned themselves as agents very early in the discussion, and spoke of their responsibility for their own wellbeing and for reaching out for help, indicated by the closed yellow loop in Figure 4. However, almost everyone – staff and students – said that they did not know what was available in their institutions in terms of mental health support, and that there was either lack of awareness, or lack of a centralised hub of information on what are often a range of dispersed services. 

Alongside this, the student-supervisor relationship was identified as a core conduit for good or poor mental health:

*‘If you do not have a good relationship with your supervisor, then you're going to have poor mental health, meaning you're probably not going to perform well in the PhD project, meaning you're not going to have good career aspirations. So, I think that this, yeah, actually finding a match is one of the most important things.’*

Many felt that the inclusion of a general mental health check-in within supervisory meetings was a useful legacy of the pandemic, which both staff and students wanted to continue.

The wellbeing of staff and students were seen to be very closely interconnected, with the stress of staff having a direct effect on students. Directors particularly noted the negative impact of current incentive structures on research culture, which filtered through to students through the supervisory relationship. The current research assessment and evaluation criteria were seen to be one of the biggest pressure points that need to be reimagined, across the whole research system, with varying levels of optimism displayed about the changes currently being made in this area. Staff members also pointed out the tensions and even contradictions in training students in a ‘kinder’ culture, which would not prepare them for the realities of actual research, as well as the demands made on academic staff to deliver both a positive research culture for students, while still being evaluated by the criteria of the existing incentive structure.

In some group discussions, we presented participants with the scenario that a student in their lab was beginning to show signs of mental health problems, asking who should notice and who should do something. The most frequent response was that supervisors and Directors should, followed by other members of the lab, such as the student cohort. Directors and supervisors felt very responsible for students’ wellbeing and mental health, with professional services staff often identified as a first port of call for students should they feel that things are going wrong. Depending on their positioning in the programme, the programme manager or administrator is often seen as a safe person to talk to as a colleague but not an academic colleague of supervisors (whereas academic colleagues were often seen to be too close to the supervisor). 

The issue of whether someone is a ‘safe’ person to talk to arises for two main reasons: firstly, because supervisors can themselves be the source of stress, and secondly, because students often suffer from fear of failure which they do not want to discuss with supervisors. Many students suffer from some form of imposter syndrome, and already feel undeserving of their place in the programme. Even if they do not initially suffer from this, laboratory life is fraught with more failure than students are used to. Students who had been particularly successful up until this point in their academic careers are now conducting research which has a high probability of failure. Supervisors were seen to be hugely important here; if supervisors’ expectations are only implicit, students can project negative judgements of their performance onto supervisors. Normalizing failure through conversation and the sharing of examples from supervisors’ own experiences were therefore seen to be hugely important for maintaining good mental health in students. Many programmes noted that they already had events and processes to facilitate these conversations in place, however students wanted to see more of these discussions and workshops. Directors particularly noted that this approach to failure was supported by the programme structure, which provided extra funding for consumables to give students “space to fail”. This was an important way in which the relationship between students with supervisors and Directors played a very important role in the quality of mental health.

Staff and students both noted that they felt a benefit of the programme structure was that there were many different people and services for students to turn to for help with their mental wellbeing. This included mentors, thesis committee members, peers, mental health first aiders, student representations, post-docs, lab members, and programme directors, amongst many others. Directors thus argued that the best way for funders to continue to act on the topic of mental health would be to continue to fund the programmes.

However, even with the best intentions, supervisors and Directors are not professionally trained in mental health. This elicited conversation about the awareness needed for self-care, *and* for taking care of others; which in in turn led to discussion of the lack of awareness of the signs of deteriorating mental health in oneself or in others, and the need for training. This is indicated by the dashed lines connecting students and supervisors with training.

A small number of participants discussed the role of family in the maintenance of mental health, with one member of staff raising this issue in the context of the pandemic. They noted the disparities between students who were able to move home to isolate with parents and those whose families were up to half the world away and impossible to visit for long periods of time. They added that families are usually unaware of the pressures of the PhD, noting that this is where universities or funders could provide support to help families better understand and help them support their wards better. Several directors also talked about the effect on mental health of family and other caring responsibilities:

*‘I had four hours sleep last night because of sick kids and trying to finish something for PhD students. So I think there is a personal cost sometimes, because I think we do put our students first. […] the honest truth is that some people over the last two years [of the pandemic] have essentially sacrificed their personal life and you know, their own well being, because of their sense of responsibility to their careers, but also to the people in their lives and their students they have to teach and everything else.’ *

### Map 3: supervisory practices

Since a central criterion of evaluation for the programme proposals was student-centred training, the funded programmes had made a commitment to it from the outset. Among the very first steps taken to meet this commitment was the directors’ choice of who to include in the supervisory pool, including deciding how to ensure that the requirements of the programmes with respect to supervisory practices were clear to everyone who joined as a supervisor (see Figure 5). Directors had a clear sense of agency – bolstered by the funding for the programmes – to influence supervisory practices, and in some cases, to change traditional supervisory styles. Simply having the funding for the PhD training gave directors the power to stipulate what was required, and in some cases to exclude those who may not be able or willing to meet those requirements. Directors noted that they would exclude those with a reputation for bullying, who made unreasonable demands, or who were what was described as ‘narcissistic’ or even ‘psychopathic’ supervisors. In general, the type of supervisor directors wanted to avoid were those who put their own interests before those of students. Directors observed that word of mouth and reputation play a large role in the exclusion of some academic staff from the pool. The processes are largely informal, and directors were generally at pains not to invite staff to be supervisors solely on the basis of numbers of students previously supervised. Indeed, in a few cases, directors had particularly focused on including early career researchers in the pool.

The student-supervisor relationship affects the degree of personalization or individualization in a student’s PhD training. Not all supervisors are able to adapt to different students, either because they have built up a set style over many years or decades of PhD training, or because of personality, or any number of other factors. The relationship also affects how students’ projects are conceived and shaped. This process can be top-down if students are perceived as needing to fit in with supervisors’ existing research projects; alternatively, the project is co-produced if there is scope for both students and supervisors to participate in shaping the project. Needless to say, greater personalization of supervision and co-shaping of research projects gives greater scope to the agency of students, and this was reflected in the discussions.

As such, discussion participants generally agreed that the match between supervisor and student was crucial in creating and maintaining a positive PhD training experience. A bad student-supervisor relationship was often directly invoked as a cause of mental health problems on the part of students, and the choice of supervisor(s) was therefore seen to be highly consequential for the quality of their PhD training experience. In making this choice, word of mouth again plays a major role, either through programme directors diplomatically steering students away from some choices, or through the opinion of other students, especially those in previous cohorts.

Reputation therefore plays a very important role in supervisor choice. The rotation year at the beginning of the Wellcome PhD training programmes, when students spend a period of time in the labs of different potential supervisors, was seen to mitigate against the role of reputation by giving students a chance to experience what it is like to work with different individuals first-hand, as well as giving them insight into the kinds of research conducted in their chosen labs.

Supervision on the programmes normally happens in teams of at least two supervisors. This means that there is at least always someone else to turn to if a student’s relationship with one supervisor is under strain. In principle, this also means that students are exposed to the supervisory practices and styles of different people, disciplines, or departments. Whether this does counterbalance a sub-optimal relationship with one supervisor depends, in part, on the trust that students have in the confidentiality of their exchanges. Therefore, supervisory teams are an important collective agent alongside any particular supervisor. Similarly placed as collective agents are thesis committees (which also play a role in the feedback process, as seen in Figure 5), whereas mentors are individual agents that are a counterpart to supervisors. Many programmes offer mentorship in some form, with the Directors often acting as mentors, having broader conversations with students not narrowly focused on their projects. When in place, this kind of mentorship means that there is a person with whom broader issues can be discussed and possibly acted on. Of course, these conversations can also inform Directors’ sense of who is likely to be a ‘good’ supervisor in a programme committed to positive research culture, although it cannot be forgotten that Directors are in turn supervisors too.

Universities, or sometimes Doctoral Schools or the equivalent, also put in place top-down procedures to structure the supervisor-student relationship. These include guidelines for supervisory practices and sometimes forms or other structured templates that supervisors and students fill in, together or individually, at the beginning and periodically throughout the supervision process. In our discussions, students felt these needed to leave more scope for individualization, with students and staff alike concerned about the utility of ‘one-size-fits-all’ forms. Students also called for more to be done outside of these forms to set formal expectations around working hours, work production, and leave. Indeed, many felt that completing such forms was often a formality, and one which was promptly forgotten about, although they appreciated that there was a formal record of their arrangement that could be used as a safety net should working hours or expectations of work production start spiraling out of control.

Things like funding security and promotion/recruitment criteria, whilst also playing a big role in the topic of mental health, were also seen to influence supervision practices, as they could dictate how a supervisor might relate to their students. Participants felt that the pressure of the incentive system driven by high impact publications might be consciously or unconsciously passed on to students through pressure to produce results or even *via* the kind of career advice offered by supervisors.

Many students and staff felt that better staff training on how to be a supervisor and how to manage supervisees would be helpful, both to help them avoid these pitfalls and to offer better guidance for staff who might be new to the position. It was felt that this kind of training could help interrupt cycles of supervision, where supervisors might repeat unhealthy practices that they themselves had been subject to during their own doctorates. Directors particularly thought that this training would be better organised on a local level, noting that they found institutional level training superficial and irrelevant. However, students expressed a desire for some standardization in training across the programmes, perhaps delivered by funders.

Students in a large number of the discussion groups also noted that they would appreciate a more robust system for providing feedback on supervision, both good and bad. Whilst they outlined informal feedback mechanisms which already existed, either within the programme ( *i.e*., speaking to peers or to programme managers), in their labs, or as part of their wider academic support (specifically their thesis committee, with varying levels of independence from the programme), many expressed a desire for a more formal system of reporting. This system, it was proposed, would report either to the funder or to the university, or even to both (as illustrated by the dashed lines extending from the feedback action in Figure 5). It could have the potential to affect the system in important ways: moving beyond word-of-mouth, reputation-based supervisor choice and moving towards a system that incentivises positive supervision, either at institutional level or funder level. Directors, on the other hand, noted that many programmes already relied on formal reporting systems within their institutions and felt that students were able to utilise these effectively. This is an important discrepancy between the views of staff and students on this issue.

Meanwhile, some noted that the pandemic had begun to produce some unexpected positive effects on the student-supervisor relationship, with many students and staff displaying heightened empathy for their counterparts ( Carusi *et al.,* 2022a). Although many would have had regular meetings with their supervisees before the pandemic, one member of staff commented that the pandemic had encouraged them to create more of a formal schedule with their students, rather than relying on informal meetings in the lab, which they had noticed had increased the amount they were meeting with his students. Under the topic of mental health, it was also noted that these meetings prompted more regular check-ins on student wellbeing (see Figure 4).

Students observed that another impact of the pandemic on supervisory practices was the introduction of more input from other staff members into the supervision process. It was noted that this was largely a result of restricted laboratory hours and reduced room capacities, which meant that they often had to turn to other members of staff for help with experiments, often solely because they were scheduled to be in the lab at the same time. Whilst supervisors retained their usual advisory role, still helping students to conceptualise and analyze their results, much of the practical work of a supervisor in the biosciences had been, by necessity, conducted by others.

Not limited to the pandemic, many noted that interdisciplinarity and collaboration were key elements of the programme structure which positively impacted research culture within these settings. Supervision in the programmes occurs in teams of two or more in order to deliver on the interdisciplinarity that most of the programmes also aspire to. Several of the programmes are particularly committed to nurturing collaboration and interdisciplinarity, which flourish with the dual impetus of a more open supervisory structure, and a more student led approach, which sees students making connections that are difficult for supervisors in single disciplines to make. This means that students ‘don’t end up in a silo from day one, it means that they’re constantly talking to other people’.

Lastly, with respect to all three maps, no matter where discussions started, they soon ranged over all of the topics we proposed for discussion, and supervision, working hours, and mental health were very interconnected. Each of the three maps shows students and supervisors to be highly interconnected with each other, as expected, but also with all the other agents. All of the other agents act on this pair of agents and thus shape or influence PhD training, while all of the other agents are in turn affected by them.

## Discussion

In our research, we have aimed to build on the research of Dhirasasna *et al.* (2021), Gibbs and Griffin (2013), and Lee *et al*. (2021) showing the aptness of a systems approach to research culture in higher education, focusing in particular on PhD training in the context of host institutions. In this section, we move from the interpretation of our data, to an hypothesis regarding what might be plausible feedback loops at play in the complex relationships that come into play in PhD training, and which may then help to identify leverage points that can be utilised for policy interventions.

**Figure 6.**
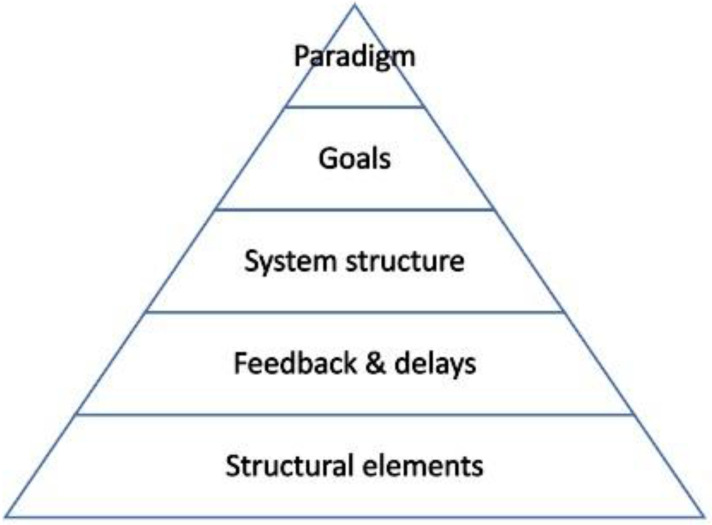
The 5-level intervention framework we have used in this study for our systems approach towards PhD training as it is situated within research culture.

Applying a systems approach levels of analysis as set out in Figure 6, we take the system to be that of PhD training as it is situated within research culture; its goal is to deliver PhD training. The paradigm is in this case the deeply held beliefs or expectations of agents with respect to how PhD training should be delivered, or what are the core values that it should express. ^4^ The paradigm informs agents’ understanding of the goals of the system but can also shift in response to that understanding. Our research shows the potential for this to occur. The goal of delivering PhD training can be understood in a number of ways: for example, A: delivering training that will enable supervisors to continue their current research activities; B: delivering training that will enable students to formulate their own projects; C: delivering training that will enable students and staff to network and collaborate in teams to generate new projects. Each of these three possible goals emerged during our discussions and interviews, with the shift in supervisory styles being seen as a shift from A to B and/or C. The new projects may not fall within the remit of any one supervisor or within the remit of any one discipline, or indeed may shift the researcher’s perspective beyond academic science altogether, to pursue a transdisciplinary or non-academic career.

**Figure 7.**
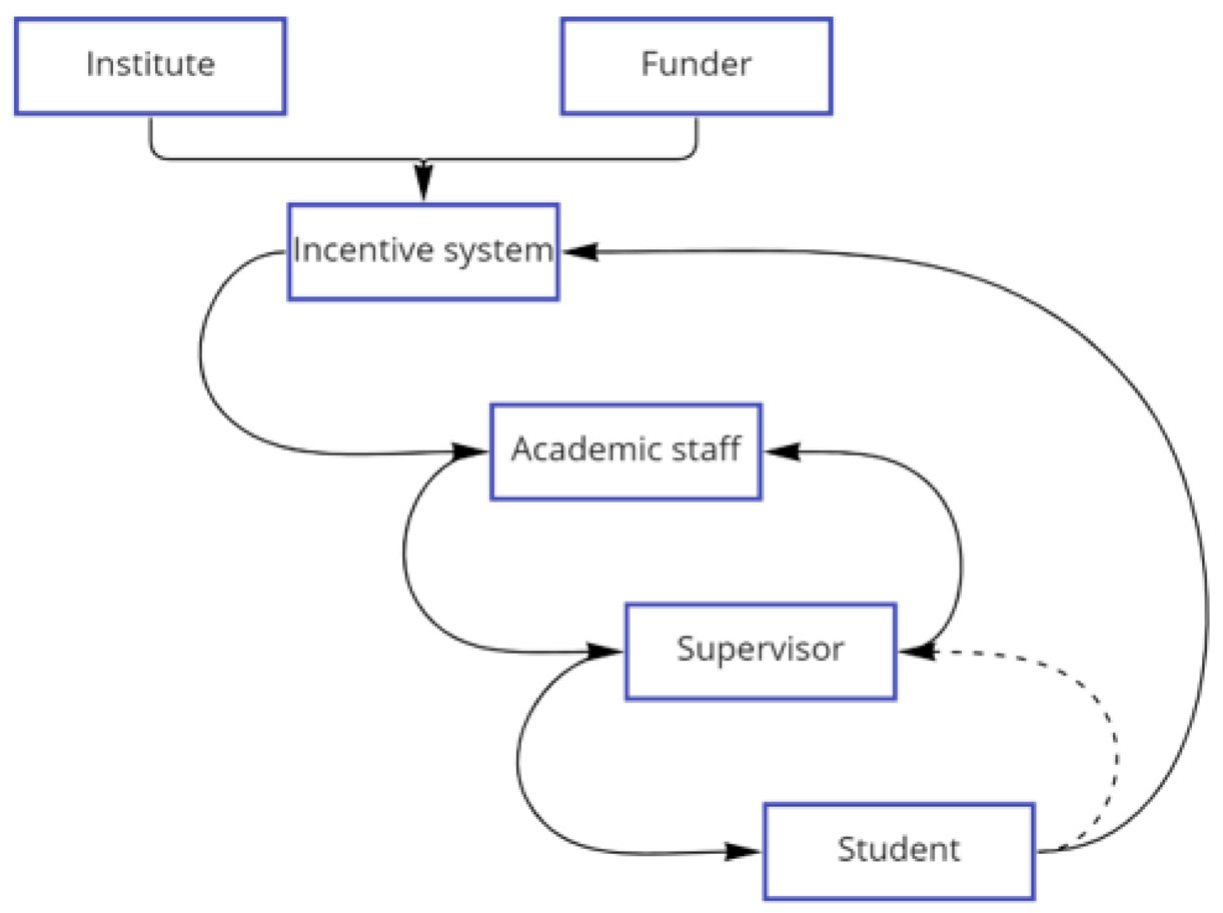
The negative feedback loop- a system which tends to be regulated by an incentive system based on productivity measured by publications in high impact journals, pressurizing the supervisors and in turn the students who then work to generate data for their supervisors (Goal A).

**Figure 8.**
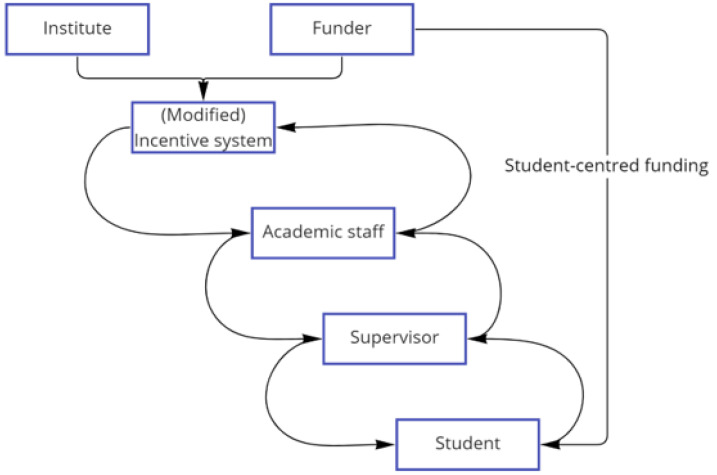
The positive feedback loop- Wellcome intervention to develop student-centred funding opportunities made it possible for students to have a stronger role in shaping their own research and its practices (Goal B).

The intervention on PhD training, requiring a student-centered approach to training, care for mental health and well-being, and support for a wide range of career transitions, acts on the structural elements of supervisors and students as agents, and the relationship between them. It acts to dislodge the feedback system which tends to shape students’ projects in accordance with the research needs of supervisors rather than students (goal A, above). This feedback system sees supervisors acting under pressure from an incentive system based on productivity measured by publications in high impact journals, and other impact, often quantitatively measured ( Figure 7). Supervisors are closely interconnected with other academic staff who are also supervisors, and often serve as internal evaluators of PhD projects, monitoring progress and ensuring quality control before external examination, and the system structure of this incentive structure permeates throughout this network. Under goal A, students’ projects are often construed as providing data for the research of their supervisors, and contributing to publications, thereby reinforcing the system. At the same time, general work ethic, or work/life routines and patterns, are also passed down through this relationship. Students in that feedback loop often model their behaviour on that of supervisors and repeat the same behaviours when they become supervisors in their turn. Wellcome’s intervention at the level of PhD training instead acted directly on the supervisor-student relationship, thereby making it possible for students to have a stronger role in shaping their own projects, as well as expectations of work ethic and work/life balance. The programmes empower them to make these demands through also empowering Directors to set requirements for standards of supervision. As they do so, the feedback from students to supervisors is strengthened; and the expectations of supervisors regarding what they and their students are entitled to in the supervisory relationship also begins to shift ( Figure 8). In virtue of the inter-relations among supervisors, reinforced by the action of Directors, this feedback loop is enhanced. It has the potential to push back on the goals of the system and to change the overarching beliefs and expectations within it so that they become more aligned with goal B.

A further way for feedback loops to bring about structural change in the system arises when students are actively encouraged to explore collaborative projects. In this case, Wellcome’s intervention is supported by interventions from the Directors and their research institutes which in turn changes the relationship among supervisors; their interconnections are more likely to be collaborative, bringing about a shift towards goal C.

The student-supervisor relationship is a strong shaping force for students; although it can also potentially be a strong shaping force for supervisors through students who are sufficiently well supported to be able to express their agency in the relationship. Support for this relationship comes from individual Directors or supervisors, but most importantly, it is institutional, in the form of the requirements of the programmes and the funding for them. The relationship between students and staff then becomes a conduit through which passes existing expectations of what supervision is (such as traditional, top-down supervisory styles), as well as a push back demand for different forms of supervision. Because of the interconnections between the students and supervisors as agents, and the strong shaping force of the relationship between them, we suggest that where a policy intervention on research culture is sought, the student-supervisor relationship is a point of leverage that could bring about changes that ripple throughout the research system. However, this needs to be supported by interventions at other levels: for example, interventions at the level of the system structure that seek to change the incentive system more directly.

We can see how this single leverage point has the potential to bring about change at the level of system structure, potentially changing the goals of the system. But what of the paradigm of the system, the deeply held beliefs about its *raison d’être*? We did not ask about this directly during our discussions and interviews, but we can form a hypothesis about what this is from the comments and observations of participants. One form of the paradigm that informs the goals of the system is the belief that students are resources for supervisors, and those who succeed will be rewarded through becoming supervisors or PIs in turn, while those who do not succeed fall out of the system. Another paradigm is that supervisors are enablers for students to meet their own potential, wherever that might take them, and that may be within the academic research system or outside of it. This is potentially a radical shift, because it could redraw the boundaries of training in scientific research. Table 1 sets out which changes can occur at the different intervention levels of the system.

**Table 1.** Changes at the different intervention level of the system (following Malhi *et al.*, 2009).

	Dominant / norm	Emerging / strengthening
Paradigm: deeply held beliefs regarding student-supervisor relationship	Students are resources for supervisors	Supervisors are enablers for students’ own career journeys.
Goal: delivering PhD training	Training that ensures students generate data for supervisors’ projects (Goal A)	Training that enables students to formulate new projects (Goal B); or enables collaboration (Goal C)
System structure	Incentive system based on high impact publications	Incentive system based on wider criteria, including quality of training; open science principles
Feedback loops	Feedback loop from students to supervisors is weak	Feedback loop from students to supervisors is strong
Structural elements	Students, supervisors and infrastructure for their relationship	

## Conclusion

Enhancing research culture is often seen as something which needs to be tackled with some urgency, and there are several ongoing initiatives which aim to intervene on this topic. In this paper, we have focused on one intervention, made by a major funder, to intervene in PhD training as a way of changing research culture more broadly. We have analyzed the system of PhD training as it is perceived by student and staff participants in the community of all funded programmes, with its main characteristics emerging during facilitated discussions and semi-structured interviews, designed to elicit understandings of agents and the interconnections between them. Our research suggests that the supervisor-student relationship plays a strong formative role on students’ experience of research culture, in terms of their expectations of working hours, mental health, and interactions with others in the environment. It is a nexus beyond the PhD training system, as it is interconnected with almost every other agent that influences research culture. Because of its positioning, changing the dynamic of this relationship has the capacity to affect other aspects of the PhD training system, including the overall paradigmatic understanding of its purpose. Beyond PhD training, it could reach into broader aspects of research culture. This is what the funder had envisaged in making this form of intervention, setting off a kind of natural experiment in these PhD programmes. Our research pinpoints the supervisor-student relationship as the pathway through which this change can happen, if it is sufficiently supported by other actions, especially on the incentive system. We suggest that this is a fruitful line of further studies on PhD training and on research culture.

## Data Availability

Our data is restricted due to data protection issues related to the inability to achieve complete anonymity within the community of practice, or outside it, as per our ethics approval. However, we have given detailed descriptions of the data in the three reports that are included in the references ( Carusi *et al.*, 2022a; Carusi *et al.*, 2022b; Carusi *et al.*, 2022c). Access to the data can be obtained by emailing a.carusi@inter-changeresearch.com, subject to agreement to the principles of confidentiality of the Wellcome Trust PhD programmes community of practice. Figshare: Materials from Emerging Research Cultures project: Directors' Thematic Interview Schedule; Themes from analysis of group discussion with PhD students, supervisors and Programme Directors, and Programme Professional Services Staff https://doi.org/10.6084/m9.figshare.23898945.v1 ( Carusi *et al*., 2023) This project contains the following extended data: Tabulated themes and codes.xlsx Directors Interview Schedule.docx Data are available under the terms of the Creative Commons Attribution 4.0 International license (CC-BY 4.0).
