# Supplementary material for: Enhancing research culture through PhD training: a systems approach to identifying leverage points for policy formation
Source: Wellcome Open Res. 2023 Sep 25;8:422. [Version 1] doi: 10.12688/wellcomeopenres.19567.1 (PMC10762290; doi:10.12688/wellcomeopenres.19567.1)
Supplement: Peer review history

# Reviewer report: Reviewer response for version 1

**DOI:** 10.21956/wellcomeopenres.21677.r70583

**Published:** 2024-01-02

**Authors:** Michelle Mae Olvido

**Affiliations:** 1 Cebu Normal University, Cebu City, Philippines

Using a systems theory methodology, this action research study focused on the potential of Ph.D. training programmes to foster a positive research culture. The researchers sought to understand agency in Ph.D. training and identified points where policy can be made to improve system conditions. Group discussions and interviews were analyzed thematically, and discussions were focused on three areas: working hours and locations, mental health, and supervisory practices. The study concludes by identifying the supervisor-student relationship as a "pathway through which change can happen."

 The minor revision I suggest to the researchers is further clarification of methods and analysis. An operationalized or contextualized definition of community of practice and a consistent use of areas looked into (e.g. mental health vs. mental health and well-being; positive research culture vs. research culture) are recommended. Furthermore, the tabulated themes are not highlighted in the map discussions, or there were a lot of discussions that cannot be traced back to the attached tabulated themes.

 The discussions shed light on every map, but it is unclear to me as a reader why these discussion points were highlighted out of the rich data generated from the discussions and interviews. What is the basis of the discussions under every map?

Is the work clearly and accurately presented and does it cite the current literature?

Yes

If applicable, is the statistical analysis and its interpretation appropriate?

Not applicable

Are all the source data underlying the results available to ensure full reproducibility?

Yes

Is the study design appropriate and is the work technically sound?

Yes

Are the conclusions drawn adequately supported by the results?

Yes

Are sufficient details of methods and analysis provided to allow replication by others?

Partly

Reviewer Expertise:

research culture, educational management, teacher education

I confirm that I have read this submission and believe that I have an appropriate level of expertise to confirm that it is of an acceptable scientific standard.

# Reviewer report: Reviewer response for version 1

**DOI:** 10.21956/wellcomeopenres.21677.r69107

**Published:** 2023-10-26

**Authors:** Sofie Bager-Charleson

**Affiliations:** 1 The Metanoia Institute, London, England, UK

I have found this a thorough, insightful and well supported text. It covers several complex stages of the study into research culture and PhD; most described well.

 I would have welcome more information about the interviews, but find that there are further earlier published sources to turn to for sufficient in-depth information about that stage too.

 The text highlights several significant issues and concerns - for instance as described in context of 'paradigms'; with contrasting aims for PhD students (supporting supervisor's research versus student's own development). The paper also expands on the 'system theory methodology' well.

 I would have welcomed a more detailed background to literature review strategy, but -again, there is sufficient information to give a good enough background to the search.

 I think thus, overall that the article, makes a significant contribution in the field.

Is the work clearly and accurately presented and does it cite the current literature?

Yes

If applicable, is the statistical analysis and its interpretation appropriate?

Yes

Are all the source data underlying the results available to ensure full reproducibility?

Yes

Is the study design appropriate and is the work technically sound?

Yes

Are the conclusions drawn adequately supported by the results?

Yes

Are sufficient details of methods and analysis provided to allow replication by others?

Yes

Reviewer Expertise:

Doctoral Research Supervision

I confirm that I have read this submission and believe that I have an appropriate level of expertise to confirm that it is of an acceptable scientific standard.
